# Ulcerative Colitis-Associated Vasculopathy Mimicking Antineutrophil Cytoplasmic Antibody (ANCA)-Associated Vasculitis

**DOI:** 10.7759/cureus.110374

**Published:** 2026-06-06

**Authors:** Sa'ed Batayneh, Anam Ansari, Mahesha Makandura, Ali Ali, Ayad Alkhatib, Amita Bishnoi, Alireza Meysami

**Affiliations:** 1 Internal Medicine, Henry Ford Health System, Jackson, USA; 2 Rheumatology, Henry Ford Health System, Detroit, USA

**Keywords:** anca-associated vasculitis, extraintestinal manifestations in inflammatory bowel disease, gangrene of the digits, microvascular thrombosis, pr3-anca, thrombotic cutaneous gangrene, thrombotic vasculopathy, ulcerative colitis (uc)

## Abstract

Ulcerative colitis (UC) is a chronic inflammatory condition with recognized extraintestinal manifestations, including a prothrombotic state that can result in both venous and arterial complications. While venous thromboembolism is more common, arterial and microvascular thrombosis leading to digital ischemia and gangrene are rare but severe manifestations. The presence of antineutrophil cytoplasmic antibodies (ANCA), including proteinase 3 (PR3) positivity, may further complicate the clinical picture by mimicking ANCA-associated vasculitis (AAV), posing a diagnostic challenge.

We present a 21-year-old male patient with a history of UC who developed rapidly progressive bilateral digital ischemia and gangrene following a recent disease flare. Laboratory evaluation revealed markedly elevated inflammatory markers and positive c-ANCA with PR3 antibodies, raising concern for AAV. Imaging demonstrated patent large vessels. Skin and renal biopsies showed no evidence of vasculitis. Despite treatment with anticoagulation, antiplatelet therapy, vasodilators, and supportive care, ischemia progressed, ultimately requiring digital and partial foot amputations. A multidisciplinary assessment supported a diagnosis of UC-associated vasculopathy after extensive evaluation excluded other causes of thrombosis.

This case highlights the importance of distinguishing UC-associated vasculopathy from AAV, particularly in the setting of ANCA positivity, which can represent a diagnostic pitfall. Early tissue biopsy is essential for accurate diagnosis and appropriate management, with a multidisciplinary approach playing a critical role in guiding care.

## Introduction

Ulcerative colitis (UC) is a chronic inflammatory bowel disease (IBD) characterized by inflammation of the colon and rectum, with well-recognized extraintestinal manifestations, which can involve the vasculature, leading to thromboembolic events [1]. Such events occur in approximately 1.3% of IBD patients; however, postmortem studies have revealed substantially higher rates, ranging from 39% to 41%, indicating that many events are clinically silent. Importantly, UC is associated with a persistent hypercoagulable state that remains present during remission and intensifies during exacerbations, consistent with case reports demonstrating a temporal association between disease flares and thrombotic events [1-6]. Presumed mechanisms of this hypercoagulable state include immune-mediated endothelial dysfunction, thrombocytosis, platelet overactivation, increased serum procoagulants and coagulation factors, activation of the coagulation cascade, and a fall in anticoagulants due to protein-losing enteropathy [1,4-8].

While venous thromboembolism (VTE) is the most common vascular complication, arterial and microvascular thrombosis, including ischemic digits and thrombotic cutaneous gangrene, have been described as rare but severe complications, reported in adults involving the trunk, extremities, digits, scrotum, and genitalia [3-13].

Antineutrophil cytoplasmic antibody (ANCA) positivity in UC

UC is classically associated with an atypical perinuclear ANCA (p-ANCA or X-ANCA) pattern in up to 70% of cases, which is distinct from the myeloperoxidase-associated p-ANCA seen in ANCA-associated vasculitis (AAV). Classical cytoplasmic ANCA (c-ANCA) is uncommon in UC. Nevertheless, recent studies have demonstrated that proteinase 3 (PR3)-ANCA may be detected in patients with UC, with reported prevalence ranging from approximately 6% by enzyme-linked immunosorbent assay (ELISA) to over 30% using high-sensitivity chemiluminescence immunoassays [14]. Such positivity represents an important diagnostic pitfall, as PR3-ANCA positivity alone does not establish a diagnosis of AAV and must be interpreted within the appropriate clinical and histopathologic context. In cases where the diagnosis remains uncertain, early tissue biopsy is essential to confirm or exclude AAV and guide appropriate management [15,16].

We report a case of UC presenting with rapidly progressive digital gangrene and positive c-ANCA/PR3 serologies, initially raising concern for AAV, in which tissue biopsy ultimately established the diagnosis of UC-associated vasculopathy.

## Case presentation

We present a 21-year-old male patient with a history of UC, vaping, and glucose-6-phosphate dehydrogenase (G6PD) deficiency. The UC was diagnosed one year prior and managed with mesalamine. At the time of diagnosis, colonoscopy with biopsies demonstrated diffuse active colitis.

The patient was transferred to our institution from an outside hospital for further rheumatologic evaluation of progressive digital ischemia and bilateral dry gangrene. Approximately one month prior to presentation, he experienced a UC flare marked by one week of hematochezia and abdominal pain, during which he self-tripled his mesalamine dose without medical supervision. Shortly thereafter, he developed numbness and tingling of the fingers and toes, with intermittent nail cyanosis. This progressed to dusky blue discoloration of the digits with worsening pain, prompting evaluation at an urgent care facility and subsequent admission to an outside hospital. Given the patient's young age, vaping history, rapidly progressive distal ischemia, and bilateral involvement, the differential diagnosis included large-vessel occlusive disease, small-vessel vasculitis, thromboangiitis obliterans (Buerger's disease), embolic phenomena, and other thrombotic vasculopathies. Computed tomographic angiography (CTA) of the bilateral upper and lower extremities and arterial and venous Doppler studies were unremarkable, making large-vessel occlusive disease less likely and increasing suspicion for small-vessel vasculitis and Buerger's disease. He was treated with intravenous methylprednisolone 40 mg every six hours, nifedipine 30 mg daily, vasodilator therapy, and multimodal pain control. Despite four days of hospitalization, his symptoms continued to worsen, prompting transfer to our institution.

**Figure 1:**
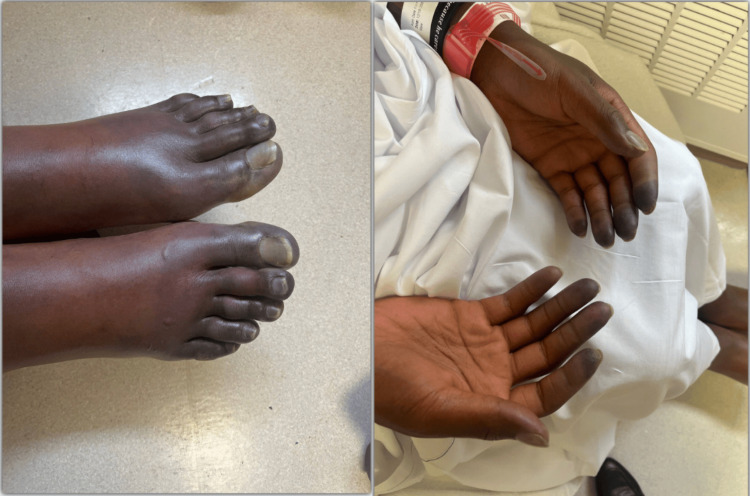
Dry gangrene involving all fingertips and toes with extension to the midfoot

On arrival, he reported severe, escalating pain and was afebrile with a temperature of 36.8°C (98.2°F), a blood pressure of 153/97 mmHg, a heart rate of 107 beats/min, a respiratory rate of 15 breaths/min, and an oxygen saturation of 95% on room air. Physical examination revealed dry gangrene involving all digits of both hands and both feet, with ischemic changes extending to the midfoot (Figure 1). The distal fingertips and toes demonstrated symmetric dusky discoloration, coolness, tenderness to palpation, and diminished but intact sensation. Radial, dorsalis pedis, and posterior tibial pulses were palpable and symmetric bilaterally.

The medications initiated at the outside hospital were continued, and an intravenous heparin infusion was started upon admission because a thrombotic vasculopathy remained a concerning diagnosis.

Laboratory evaluation (Table 1) revealed markedly elevated inflammatory markers, including an erythrocyte sedimentation rate (ESR) of 103 mm/hour (normal range: <15 mm/hour) and a significantly elevated C-reactive protein (CRP) of 23.4 mg/dL (normal range: <0.5 mg/dL). He was also found to have anemia (hemoglobin: 9.6 g/dL), a platelet count of 239 K/uL, leukocytosis (31.9 K/uL), elevated lactate dehydrogenase (570 U/L), low haptoglobin, and total bilirubin of 1.9 mg/dL. Although the white blood cell count was markedly elevated, the patient remained afebrile, blood cultures were negative, and imaging failed to identify an infectious source, making active inflammation related to the UC flare and corticosteroid therapy the most likely contributors. Urinalysis showed non-nephrotic-range proteinuria with hematuria and an elevated urine protein-to-creatinine ratio of 1.55 which nearly normalized on subsequent testing. No red blood cell casts were identified. Direct antiglobulin testing was negative, arguing against immune-mediated hemolysis. Fibrinogen levels were elevated, but coagulation studies were not consistent with disseminated intravascular coagulation. Antiphospholipid antibody testing demonstrated negative anticardiolipin and β2-glycoprotein I antibodies, making catastrophic antiphospholipid syndrome unlikely; lupus anticoagulant testing was uninterpretable due to therapeutic anticoagulation.

**Table 1:** Laboratory results CBC: complete blood count; ESR: erythrocyte sedimentation rate; CRP: C-reactive protein; ANA: antinuclear antibody; c-ANCA: cytoplasmic antineutrophil cytoplasmic antibody; PR3: proteinase 3; p-ANCA: perinuclear antineutrophil cytoplasmic antibody; MPO: myeloperoxidase; ASMA: anti-smooth muscle antibodies; aHUS: atypical hemolytic uremic syndrome; CCP: cyclic citrullinated peptide; dsDNA: double-stranded DNA; PT: prothrombin time; INR: international normalized ratio; PTT: partial thromboplastin time; RBC: red blood cells; HPF: high-power field; Cr: creatinine

Category	Test	Result	Reference range
CBC	Hemoglobin (g/dL)	9.6	13.5-17
	White blood cells (K/uL)	31.9	3.8-10.6
	Platelet count (K/uL)	239	150-450
	Haptoglobin (mg/dL)	<30	30-200
Chemistry/inflammatory	Total bilirubin (mg/dL)	1.9	<1.2
	Direct bilirubin (mg/dL)	0.7	0-0.3
	ESR (mm/hr)	103	<15
	CRP (mg/dL)	23.4	<0.5
Autoimmune studies	ANA	Negative	<1:80
	c-ANCA	1:80	<1:20
	PR3 antibody	2.8	<2
	p-ANCA	Negative	Negative
	MPO antibody	Negative	Negative
	ASMA (units)	22	<20
	Direct antiglobulin	Negative	Negative
	ADAMTS13	Negative	Negative
	aHUS	Negative	Negative
	β2-Glycoprotein IgA, IgM, IgG	Normal levels	Negative
	Cardiolipin IgA, IgG, IgM	Normal levels	Negative
	Centromere antibody	Negative	Negative
	CCP IgG	Negative	Negative
	dsDNA	Negative	Negative
	SCL-70 antibody	Negative	<7 U/mL
Coagulation studies	PT (sec)	14.8	11.5-14.5
	INR	1.13	-
	PTT (sec)	29	22-36
	Fibrinogen (mg/dL)	965	200-450
Urine studies	Urine protein (mg/dL)	100	Negative
	Urine blood	+1	Negative
	Urine RBCs (HPF)	13	<3
	Urine protein/Cr ratio (mg/mg)	1.55	<0.15

C-ANCA was positive at low titer (1:80) with PR3 antibody positivity. In the setting of progressive digital ischemia, elevated inflammatory markers, and urinary abnormalities, these findings raised concern for AAV and prompted further evaluation for systemic involvement, including additional imaging studies and plans for tissue biopsy. Additional autoimmune serologies were negative.

CTA of the upper and lower extremities demonstrated patent large arteries without vessel wall thickening, flow-limiting stenosis, luminal irregularity, or intraluminal thrombus. Arterial Doppler studies showed normal ankle-brachial, toe-brachial, wrist-brachial, and finger-brachial indices with triphasic or multiphasic waveforms throughout. Collectively, these findings argued against large-vessel occlusive disease.

A high-resolution computed tomography (CT) scan of the chest was done, showing ground-glass opacities. Pulmonology was consulted for suspicion of autoimmune processes, but given that the patient has been on high-dose steroids, the suspicion was low.

Renal ultrasonography showed normal kidney size and echogenicity. Transthoracic echocardiography revealed no intracardiac thrombus or valvular abnormalities, potentially ruling out embolic cardiac etiologies.

**Figure 2:**
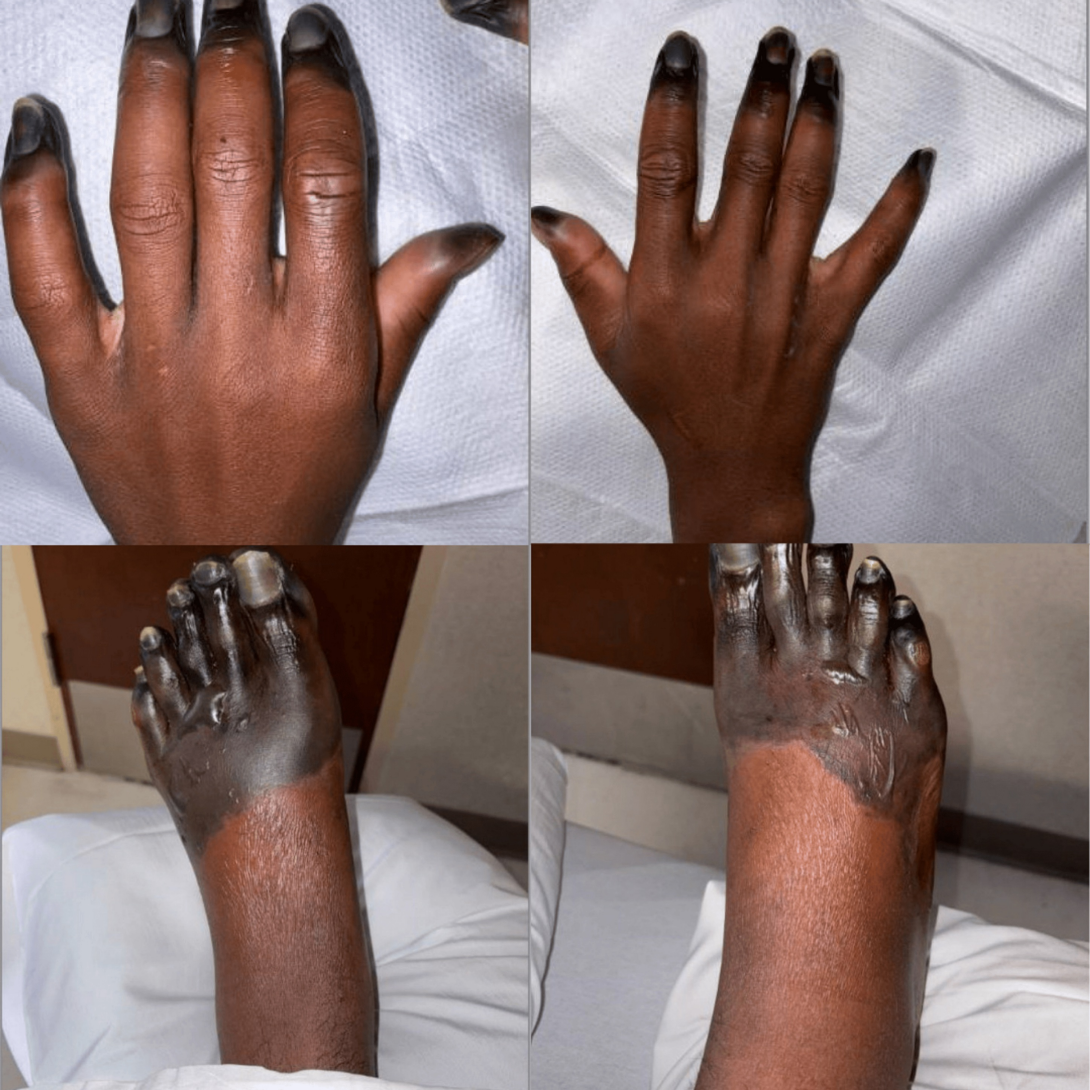
Worsening and proximal progression of the dry gangrene in the bilateral hands and feet with blistering formation in the feet

Following the ANCA positivity, on the third day of admission, pulse-dose intravenous methylprednisolone (1 g daily) was initiated, followed by prednisone at 1 mg/kg daily. But, despite aggressive medical management, ischemia progressed to symmetric dry gangrene with blistering new areas of black discoloration on the bilateral heels of the patient's feet (Figure 2).

The onset of urinary abnormalities after the initiation of intravenous heparin made the findings less consistent with ANCA-associated glomerulonephritis, particularly given the absence of red blood cell casts. There was insufficient evidence to support a diagnosis of AAV, and we cannot definitively rule out kidney involvement without a biopsy, especially since the patient had been on steroids for several days. Therefore, we recommended obtaining tissue biopsies of the skin and kidney.

**Figure 3:**
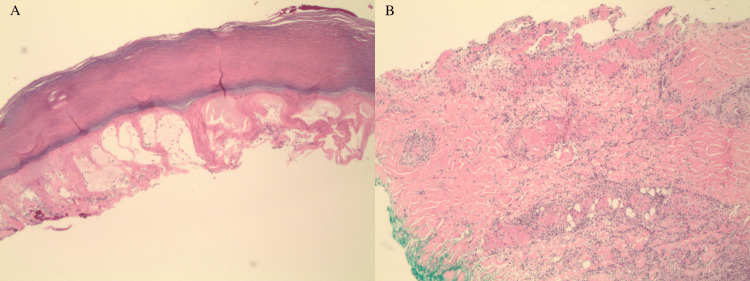
Punch biopsy of the right foot (A) Full-thickness epidermal necrosis with no attached dermis (H&E, ×40). (B) Focal ulceration with mixed inflammation and multiple small vessels demonstrating occlusive thrombosis, concerning for vasculopathy (H&E, ×40).

**Figure 4:**
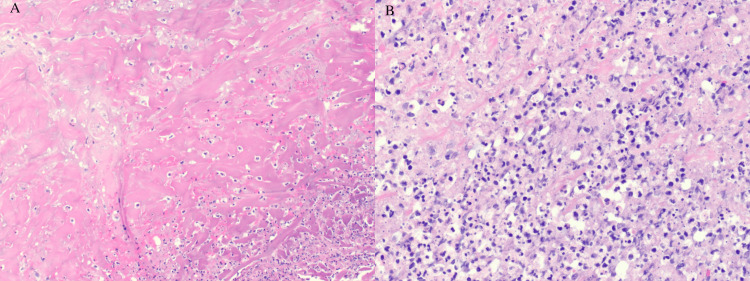
Right and left partial foot amputation biopsy (A-B) Focal ulceration, gangrenous necrosis, and acute inflammation without evidence of fibrinoid necrosis, leukocytoclasia, or vasculitic vessel wall injury (H&E, ×40).

Skin biopsy of the right foot revealed epidermal necrosis with underlying fibrosis and small-vessel thrombosis, without evidence of vessel wall inflammation or necrotizing vasculitis, which can be seen in AAV (Figures 3-4).

**Figure 5:**
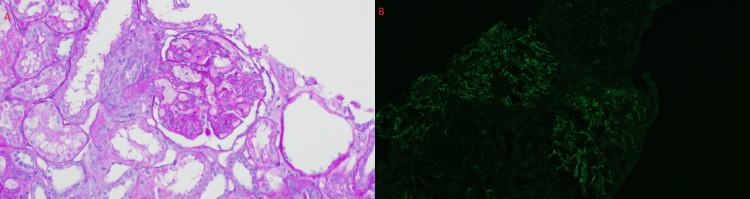
Renal biopsy (A) Light microscopy showing endocapillary hypercellularity with fibrin thrombi and mesangiolysis, without fibrinoid necrosis or crescent formation to suggest vasculitis (H&E, ×40). (B) Immunofluorescence microscopy demonstrating diffuse granular mesangial C3 deposition in glomeruli.

Renal biopsy demonstrated thrombotic microangiopathy-like changes with focal proliferative and exudative C3-dominant glomerulonephritis, interpreted as secondary ischemic injury rather than primary pauci-immune glomerulonephritis (Figure 5).

Hematology was consulted given concern for vasculopathy and hemolytic anemia. They concluded that any possible hemolysis was most likely attributable to the patient's underlying G6PD deficiency and that his clinical and laboratory findings were not consistent with microangiopathic hemolytic anemia (MAHA) or thrombotic microangiopathy. Peripheral blood flow cytometry revealed no clonal hematologic disorder. Testing for paroxysmal nocturnal hemoglobinuria was negative, pyruvate kinase activity was adequate, and ADAMTS13 activity was not consistent with thrombotic thrombocytopenic purpura. Collectively, these results excluded primary thrombotic microangiopathy, hemolytic uremic syndrome, clonal hematologic disorders, immune-mediated hemolysis, disseminated intravascular coagulation, and catastrophic antiphospholipid syndrome.

Multidisciplinary team discussion favored a diagnosis of UC-associated vasculopathy driven by inflammatory hypercoagulability. Due to progressive tissue necrosis and rising concern for secondary infection, the patient ultimately required digital and partial foot amputations. The patient was quickly tapered off the steroids and discharged with aspirin and apixaban for long-term anticoagulation.

## Discussion

This case shows the difficulty of distinguishing UC-associated vasculopathy from AAV. Although the patient's presenting symptoms along with c-ANCA and PR3 positivity, elevated inflammatory markers, and urinary abnormalities initially raised concern for AAV, multiple clinical, laboratory, and histopathologic features argued against this diagnosis. Isolated cutaneous involvement in AAV is exceedingly rare, with digital ischemia and gangrene reported in fewer than 1% of granulomatosis with polyangiitis (GPA) cases and almost always occurring in the context of multiorgan disease. The absence of systemic vasculitic features in this patient, therefore, made AAV unlikely. Therefore, premature initiation of vasculitis-directed immunosuppression such as rituximab was avoided as it carries significant risks, including serious infections [17]. In our case, the biopsies demonstrated microvascular thrombosis without vasculitis in the skin and thrombotic microangiopathy-like ischemic changes in the kidney, excluding pauci-immune glomerulonephritis.

Clinical approach while awaiting biopsy confirmation

Management of such cases is challenging and requires a multidisciplinary approach. Early tissue biopsy is important for establishing a definitive diagnosis and should be obtained upon presentation, along with a hypercoagulable evaluation [6,7]. While awaiting histopathologic confirmation, treatment with corticosteroids, antiplatelet therapy (e.g., aspirin), anticoagulation with heparin, and vasodilators to improve distal perfusion should be initiated [1,4,6]. This approach allows for early management of inflammation-induced hypercoagulability while avoiding premature escalation to vasculitis-directed immunosuppression in the absence of definitive tissue diagnosis. Long-term prevention of recurrent thrombotic events likely also depends on effective control of the underlying intestinal inflammation.

Given the chronic prothrombotic state associated with UC [3], plans for long-term anticoagulation were made. Despite aggressive medical management, ischemic injury progressed to irreversible gangrene. Surgical amputation was ultimately required to remove nonviable tissue and reduce the risk of secondary infection. Total colectomy may be required in treatment-resistant cases as supported by a reported case of UC complicated by thrombotic cutaneous gangrene with penile autoamputation. In that case, colectomy was done followed by multiple excisions and grafting [7].

## Conclusions

UC is associated with a basal prothrombotic state that can worsen during flares and result in severe vascular complications. Effective control of the underlying intestinal inflammation through the optimization of UC-directed therapy is therefore an important component of preventing recurrent thrombotic events. Clinicians must maintain a high index of suspicion for these rare, catastrophic complications. ANCA positivity alone should not be considered diagnostic of a vasculitic process in the absence of supportive clinical and histopathologic findings; therefore, early tissue biopsy is essential to guide management and should be obtained before the escalation of immunosuppressive therapy.
